# Creutzfeldt-Jakob disease: A case report

**DOI:** 10.22088/cjim.12.0.359

**Published:** 2021

**Authors:** Razieh Salehian, Farzad Sina, Sussan Moudi

**Affiliations:** 1 Mental Health Research Center, Rasoul-e-Akram Hospital, Iran University of Medical Sciences, Tehran, Iran; 2 Department of Neurology, Rasoul-e-Akram Hospital, Iran University of Medical Sciences, Tehran, Iran; 3 Social Determinants of Health Reseach Center, Health Research Institute, Babol University of Medical Sciences, Babol, Iran

**Keywords:** Creutzfeldt-Jakob disease, Cognitive decline, Neurodegenerative disorders

## Abstract

**Background:**

Creutzfeldt-Jakob disease (CJD) as a life-threatening neurodegenerative disorder is not usually diagnosed in early stages of the disease because of a variety in its clinical manifestations. This study aimed to present a middle-aged woman with psychiatric symptoms who ultimately was diagnosed as a CJD case.

**Case presentation:**

This 48-year-old woman had progressive symptoms of depressed mood, decreased sleep and appetite and mutism which started two months before the first visit. Gradually, slowness in movements, dysarthria and decreased performance were observed. Subsequently, when antidepressant and antipsychotic drugs were prescribed other symptoms such as ataxia and rigidity manifested in the patient. The problem list which led to final confirmation of the disease included non-specific neuropsychological presentations, hypersignality in caudate and putamen areas in brain MRI, generalized high frequency sharp waves in EEG, and protein 14-3-3 identification in cerebrospinal fluid.

**Conclusion:**

Although CJD is not a common disease, it should be considered in differential diagnoses whenever neuropsychological manifestations, especially progressive decline in cognition, along with symptoms such as visual hallucinations, myoclonus and ataxia are observed in the patient.

Creutzfeldt-Jakob disease (CJD) is a life-threatening neurodegenerative disorder which is caused by an abnormal form of host-encoded proteins named prions (1). Cognitive impairment is the most common initial presentation of this disease and may be observed in 35% of patients (2). Since there is a great variation in the symptoms of the disease, diagnosis of CJD is always challenging, especially in the early stages (4, 5). Four subtypes of CJD have been identified: familial, iatrogenic, variant and sporadic. The sporadic form is the most common subtype of the disease and accounts for 85% of CJD cases (2, 6). CJD (sCJD) is one of the differential diagnoses of dementia (3). The incidence of CJD is one case in per million people a year and it is estimated that 350 cases are diagnosed in the United States annually (6). Although CJD is a rare disease, it has increased in recent years in some areas, for example, in Europe (4, 7) and Slovenia (2). This study was aimed to present a case referred by psychiatric symptoms and ultimately CJD was confirmed in this patient.

## Case presentation

A 48-year-old woman, married with a below high school diploma educational level, a housekeeper, living in a northern city of Iran, who has suffered from some symptoms including depressed mood, loss of appetite, insomnia, and mutism that started two months ago without any significant stressor. 

Some medications including fluphenazine decanoate, venlafaxine, sodium valproate, duloxetine, olanzapine and trihexyphenidyl had been prescribed by a neurologist in an outpatient clinic. Gradually, in addition to ataxia, slowness of movements, dysarthria, rigidity, uncontrollable movements of the limbs, and loss of performance occurred to the extent that she was unable to do her own household chores. Sometimes, she stares at a point and screamed horribly. Following the receipt of modecate, symptoms such as rigidity have exacerbated; and due to the decreased levels of consciousness, the patient was referred to the emergency department of a general hospital; thereafter the patient´s treatment was interrupted and she was referred to a psychiatric hospital following consultation by a psychiatrist. In the psychiatric ward, considering the patient's cooperation, cranial examinations were normal. Muscular strength was symmetrical and paratonia was observed in all four extremities. 

Deep tendon reflexes (DTR) was 2+. Serum therapy and urine catheterization was conducted to modify the patient´s inadequate fluid intake and low urine volume. Counseling with a cardiologist and anesthesiologist was performed to start electroconvulsive therapy (ECT) for the patient. Diazepam was administered four times before the ECT. Regarding the diagnosis of catatonia, the patient was administered two ECT. Paraclinical assessments showed some abnormal findings including leukocytosis (WBC= 12600, PMN=70%), CPK=1129, LDH=723, and P=4.7. Other routine tests including thyroid function tests, sodium, potassium and liver function tests were in normal range; and the diagnosis of malignant neuroleptic syndrome (NMS) was made due to long-term antipsychotic medications, rigidity, fever, and elevated CPK and LDH. 

She was referred to a subspecialized hospital for further investigation and the probability of NMS. In the general examination, the apparent age was greater than the chronological age and self-care was impaired. The most important findings were 38ºC fever, mydriatic and reactive pupils to the light with inability to follow the movements of the hand, lack of visual and verbal communication, rigidity, negativism and echolalia. The patient did not have a history of organ transplantation and had no occupational exposure to livestock and raw animal products. In the past medical history, she reported being referred to a psychiatrist in 1988 because of distress and depressive symptoms following a stressor which was treated with drug use for three years. The history was negative for other psychiatric disorders. She underwent splenectomy following a car crash 17 years ago. A positive family history of depressive symptoms was reported in her mother who was under treatment for many years. No cognitive and motor symptoms were reported in her close relatives. 

**Figure 1-2:**
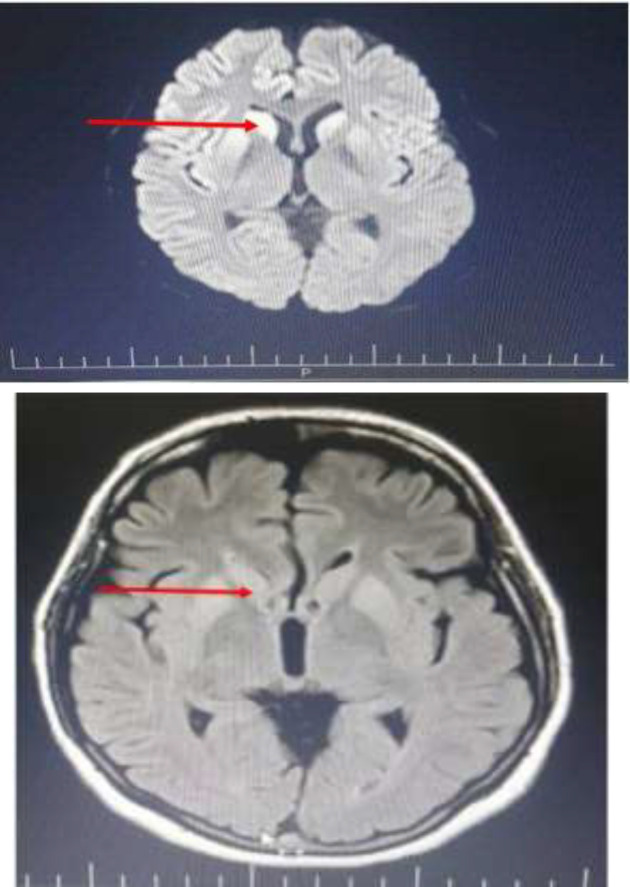
Brain MRI (Hyperintense signal in right and left head of caudate and putamen)

Routine analysis of lumbar puncture was normal. Laboratory tests for inflammatory and some infectious diseases (including Herpes Simplex Virus 1, 2, Cytomegalovirus, Epstein-Barr Virus, Varicella-Zoster Virus, Enterovirus, Human Herpesvirus VI, VII, VIII) were negative. In brain MRI, hypersignality in caudate and putamen has been reported (figures 1, 2). 

**Figure 3:**
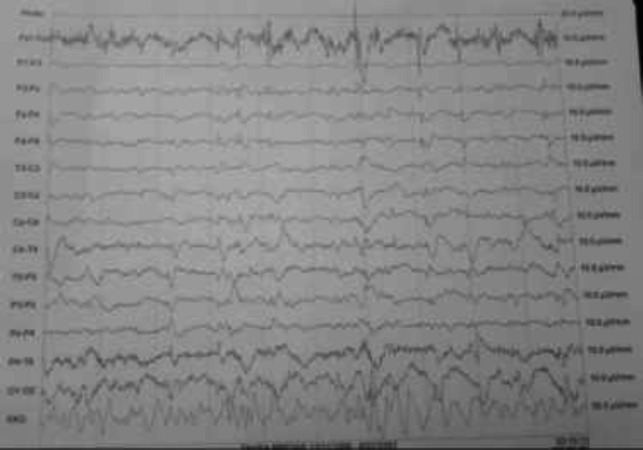
EEG (Frequent periodic burst of generalized sharp activity every one second)

Generalized high frequency sharp waves were observed in EEG (figure 3). The patient was admitted to ICU because of decreased consciousness, and levebel 500 mg BID and sodium valproate 400 mg BID was prescribed to resolve muscle spasms and myoclonus of the patient which was associated with reduced symptoms. Following evaluations, protein 14-3-3 was identified in the cerebrospinal fluid, and the diagnosis of CJD was confirmed in the patient.

## Discussion

The patient was diagnosed as a CJD case because of neuropsychological presentations (such as mutism, ataxia, myoclonus), brain MRI findings, EEG, and protein 14-3-3 identification in cerebrospinal fluid. WHO hasefined the diagnostic criteria of sporadic CJD comprising progressive dementia, with at least two out of the four clinical signs (myoclonus, visual or cerebellar impairment, pyramidal or extrapyramidal dysfunction, and atypical mutism) along with atypical EEG pattern in the course of the disease and identification of protein 14-3-3 in cerebrospinal fluid (8, 9). CJD patients usually are presented at first with non-specific behavioral abnormalities including anxiety, asthenia, depressive symptoms, loss of appetite, change in sleep pattern, weight loss, fatigue, confusion, and agoraphobia. Most of these patients may suffer from amnesia, disturbed cortical function, such as reasoning, calculation, judgment, and so on. 

One third of these patients initially are manifested with neurological disorders, including cerebellar imbalances; and a small proportion of patients are presented with a combination of cognitive impairment and focal neurological symptoms. With disease progression, dementia becomes more severe in patients, aphasia or apraxia may appear; and choreiform-athetoid movements, myoclonus jerks, and pyramidal or extrapyramidal signs may be observed (9). The diagnosis of CJD is always accompanied by challenges, as various symptoms, especially in the early stages of the disease, may be present in the patient, and many of these symptoms may be similar to other forms of dementia (4). Mackenzie, in his study, introduced sporadic CJD as a disorder with a very rapid duration, with a mean survival rate of 6 months; and more than 90% of patients die within one year of the onset of symptoms. A hypothesis about this disease represents that sporadic CJD is a spontaneous neurodegenerative disorder which occurs either from the mutation of the PRNP gene or by a random structural change in the PrP protein and causes the formation of PrPsc. The environmental source of this type has not been confirmed in epidemiological studies (7). 

Behaeghe systematic review, reveals that although autopsy is the gold standard for CJD diagnosis, the measurement of protein 14-3-3 in cerebrospinal fluid is recognized as an important low-invasive technique. In this study, the diagnostic value of protein 14-3-3 in the cerebrospinal fluid was introduced with sensitivity and specificity of 88% and 80%, respectively (3). In the patient´s brain MRI, hypersignality was observed in caudate, and putamen. In the Mackenzie´s review, high-signal pattern in the caudate, putamen, or cortex (or a combination of these) has been introduced as a significant finding of brain MRI in patients with sporadic CJD (7). In Caobelli's research, the role of different neuroimaging techniques in CJD patients was reviewed. According to this study, the percentage of people diagnosed with MRI findings alone was much more common in comparison with the CJD patients diagnosed using PET, PET + MRI or SPECT. The authors found brain MRI as the most extensive neuroimaging technique used in the diagnosis of CJD patients. They explained since the variety of radiographic findings is seen in CJD patients, it is not possible to describe a typical pattern for this disease, but the majority of patients have cortical involvement and changes in basal ganglia. Among the cortical areas, frontal (46.4%) and parietal (40.6%) lobes were the most commonly encountered areas (4). In this patient, generalized high frequency sharp waves have been shown to be an important electroencephalographic finding. In the Sitammagari’s study, EEG was introduced as a method with the least diagnostic sensitivity (compared with brain MRI or CSF fluid evaluation), and periodic sharp wave complexes were reported as the most common EEG finding in CJD patients (6). 

The disease has been manifested in a 48-year-old woman. Sitammagari represented the peak age of sporadic CJD in 55-75 with an average age of 61 years (6). Rus also introduced nine CJD patients, mostly female (six of nine), and the mean age of patients was 71.6±10.0 years (2). Mackenzie reported the peak age in the 70s and declared that the disease is less common in the age group of 20-40 or older than 80 (7). The clinical manifestations of our patient were primarily psychological and were not distinguishable from the common psychiatric disorders. In the past, sCJD was mostly characterized by neurological symptoms, but a retrospective review of 126 cases of sCJD showed that 80% of the patients during the first 100 days of the disease had psychological symptoms including depression, anxiety, psychosis, behavioral changes, and sleep disorders and 26% of them had these symptoms at the time of the medical examination (10). In this patient, catatonia has been identified initially. Milanlioglu also introduced the CJD patient, whose catatonic depression was a dominant clinical presentation (11). It is recommended that the evaluation of patients with catatonia should not be limited to depressive disorders and psychiatric disorders; however, other general medical etiologies and diseases, including CJD, should be followed-up. 

There is wide differential diagnosis for CJD, including vascular disorders (12), neurodegenerative, autoimmune, infectious, thromboembolic, malignant metastatic, iatrogenic, toxic or metabolic conditions that can result in rapid progressive dementia or progressive cognitive impairment (6). In conclusion although CJD is not a common disease, it should be considered in differential diagnoses whenever neuropsychological manifestations, especially progressive decline in cognition, along with symptoms such as visual hallucinations, myoclonus and ataxia are observed in the patient.
